# Perceived motivational climate, doping attitudes, and doping temptation among elite adolescent athletes: The moderating role of perfectionism

**DOI:** 10.1002/ejsc.12244

**Published:** 2025-02-17

**Authors:** Jan Åge Kristensen, Maria Kavussanu, Yngvar Ommundsen

**Affiliations:** ^1^ Department of Sport and Social Sciences Child and Youth Sport Research Center Norwegian School of Sport Sciences Oslo Norway; ^2^ School of Sport Exercise and Rehabilitation Sciences University of Birmingham Birmingham England

**Keywords:** attitudinal, motivational climate, perfectionism, youth sport

## Abstract

A sports culture that emphasizes normative success, as evident in a performance motivational climate, may encourage athletes to use prohibited performance‐enhancing substances. Athletes with perfectionistic tendencies are believed to be particularly tempted to doping when finding themselves in such sporting environments. In this study, we examined whether perceived motivational climate was related to doping temptation directly and indirectly via doping attitudes and whether perfectionism moderated this indirect relationship. The sample comprised 420 adolescent athletes aged 16–18 years (mean age = 16.94 and standard deviation = 0.81) recruited from five Norwegian sport academy high schools. Regression analysis revealed that athletes' perceptions of a performance climate were positively related to their temptation to dope both directly and indirectly via doping attitudes, and that this indirect relationship was stronger among athletes who were moderate or high in their perfectionistic concerns. No direct or indirect relationships were found between mastery climate and doping temptation, nor did perfectionistic strivings moderate the indirect relationship between mastery climate and doping temptation via doping attitudes. Taken together, our findings suggest that athletes who perceive their sport environment as performance‐oriented and believe that the benefits of using prohibited substances outweigh the drawbacks are more tempted to dope. Moreover, this tendency is particularly notable among athletes who are moderately or strongly concerned about making mistakes (i.e., have perfectionistic concerns).

## Highlights

- Performance climate is positively related to doping temptation both directly and indirectly via doping attitudes.
- Mastery climate is not related to doping temptation or doping attitudes.
- Perfectionistic concerns moderate the indirect relationship between performance climate and doping temptation via doping attitudes.
- Athletes with high and moderate levels of perfectionistic concerns may exhibit more positive attitudes toward doping than athletes with low levels of concerns when experiencing the climate to be performance‐oriented.

## 1 INTRODUCTION

In their pursuit of excellence in sport, athletes often use various substances and methods to enhance performance, facilitate recovery, and prevent illness (Maughan et al., 2018). According to the World Anti‐Doping Agency (WADA), some substances and methods are problematic and thus prohibited from use in sports collectively categorized as doping (WADA, 2021). Examples of these substances include anabolic steroids, hormones, and amphetamines, which arguably, have the potential to compromise the health of athletes (Birzniece, 2015; Obasa & Borry, 2019). Despite WADA's effort to promote and coordinate adherence to anti‐doping rules, the number of athletes resorting to the use of doping is rising (Gleaves et al., 2021). With the increasing prevalence of doping follows a growing interest in primary interventions that will reverse this trend before it finds its way into youth sporting cultures (Pöppel, 2021; Pöppel & Büsch, 2022).

Because it involves breaking the rules of sport, doping is widely regarded as a form of cheating and is believed to be more prevalent in sporting environments characterized by interpersonal competition, social comparison, and public evaluation (Kavussanu et al., 2020). There is also evidence that athletes' attitudes toward doping play an important role in their vulnerability to dope (Lazuras et al., 2015; Madigan et al., 2016), and that sport environments encouraging athletes to view their achievements as a matter of individual development may have the potential to discourage athletes from doping (Guo et al., 2021). However, existing research on doping has yet to assess doping temptation in young athletes while capturing the dynamic interplay between the personal factors of the athletes and the contextual factors summarized from athletes' self‐described perceptions of the sporting environment (Kristensen et al., 2022).

Numerous theoretical models have been commonly used to shed light on the psychosocial processes influencing athletes' decisions to use doping, including the achievement goal theory (Nicholls, 1989), the theory of planned behavior (Ajzen, 1991), and the sport drug control model (Donovan et al., 2002), which later was adopted for the context of youth sport (Nicholls et al., 2014). However, these models emphasize the immediate or prospective influences of personal factors that are partially within an individual's control, such as attitudes and self‐esteem, and may neglect other important influences on behavior being more external to the individual (Fishbein & Cappella, 2006). To extend previous research and enable a more complex understanding of the dynamic interplay between personal and contextual factors, we took advantage of a social cognitive approach by utilizing an integrative model (Fishbein, 2009). Adhering to a multitheoretical framework (Ajzen, 1991; Ames, 1995), we examined the relationships between motivational climate, attitudes toward doping, perfectionism, and doping temptation among adolescent athletes.

### 1.1 Perceived motivational climate

The social context within the sporting environment might enable athletes to indulge in various behaviors to better their athletic performances. According to Ames (1995), the motivational climate refers to the goals and behaviors emphasized and the salient values in the social environment created by significant others such as coaches, peers, and parents. The significant others determine what are important achievement criteria, and via their behavior they communicate what they value in a specific context and the criteria they use to evaluate success. Coaches create a *performance* motivational climate when they define success as winning, reward only the best performer, and place emphasis on outperforming others, and conversely, a *mastery* motivational climate when they encourage effort and cooperation, emphasize learning, and personal improvement (Ames, 1992). Athletes who perceive the motivational climate of their team as predominantly performance‐oriented may be more tempted to use banned substances to establish superiority over others. In contrast, a predominantly mastery‐oriented climate may encourage athletes to view their achievement as a matter of individual development, take the edge of environmental pressure emphasizing social comparison standards for success, and thus help reduce athletes' temptation to dope.

Previous studies have supported the link between performance climate and doping variables in sports (Guo et al., 2021; Kavussanu et al., 2020). For example, Guo et al. (2021) reported that performance climate was positively related to doping intentions among adolescent athletes from various individual and team sports (e.g., athletics, cycling, swimming, and rugby). Football players who perceived a performance motivational climate in their team were more likely to use banned substances to enhance their performance (Kavussanu et al., 2020). There is also evidence suggesting that a performance climate may lead young athletes to a lack of respect for rules, officials, and social conventions (Ommundsen et al., 2003). The latter finding underlines that a performance climate may elicit inappropriate or dysfunctional behavioral patterns typically associated with doping (Kristensen et al., 2022).

Mastery climate may act as a buffer for doping. For example, Guo et al. (2021) reported that mastery climate was negatively related to doping intentions. There is also evidence suggesting that young athletes who perceive the motivational climate as predominantly mastery‐oriented report more mature moral reasoning and better sportspersonship behaviors (Ommundsen et al., 2003). Nonetheless, the evidence empirically linking mastery climate to doping behaviors is not as strong. Taken together, previous research suggests that a coach‐created performance motivational climate should be considered a risk factor for doping, whereas the opposite might be expected for a mastery climate.

### 1.2 The mediating role of doping attitudes

Apart from examining the role of athletes' perceptions of the motivational climate on doping, it is also important to understand the psychological processes through which the motivational climate may lead to doping. One central antecedent of doping is attitudes, which has been defined as a psychological tendency that is expressed by evaluating a particular entity with some degree of favor or disfavor (Eagly & Chaiken, 1993). Attitudes are multidimensional in structure, composed by cognitive, affective, and behavioral components, and thus believed to guide athletes' decision about whether to engage in doping (Conner & Sparks, 2015). For example, an athlete who believes that using prohibited substances will lead to more positive than negative consequences is expected to favor doping.

Several studies have confirmed a positive relationship between doping attitudes and doping behavior (Barkoukis et al., 2014; Chan et al., 2015; Lazuras et al., 2015). In a recent meta‐analysis, Ntoumanis et al. (2024) listed attitudes toward doping as one of the strongest predictors of both doping intentions and use. Barkoukis et al. (2014) and Chan et al. (2015) reported that athletes were more susceptible to dope when they believed doping to be personally beneficial. Furthermore, attitudes are also believed to be adaptive responses to environmental demands, shaped by current accessible information and specific characteristics of the social environment (Ajzen, 1991; Schwarz, 2007). For example, social environments characterized by intrateam rivalry and unequal recognition are likely to reinforce athletes' beliefs that using prohibited substances would lead to more positive than negative consequences.

Previous research has provided support for the link between motivational climate and attitudes toward doping (Allen et al., 2015; Guo et al., 2021). Guo et al. (2021) found that adolescent athletes' perceptions of a performance climate were linked to more positive evaluations on the potential benefits of doping use such as being more confident of winning. In contrast, Allen et al. (2015) reported that competitive Scottish athletes' perceptions of a mastery climate were negatively related to prodoping attitudes. Taken together, previous research suggests that athletes' attitudes may serve as a mediator in the relationship between their perceptions of the motivational climate and their temptation to dope. Specifically, a performance climate may increase athletes' temptation to dope by eliciting prodoping attitudes, whereas a mastery climate may prevent doping by decreasing doping attitudes.

### 1.3 The moderating role of perfectionism

The relationship between athletes' perceptions of the motivational climate in their team and their attitudes toward doping may be moderated by personality traits, one of which is perfectionism. According to Frost et al. (1990), perfectionism is broadly defined as a multidimensional personality trait characterized by a combination of excessively high personal standards and overly critical self‐evaluation. Furthermore, perfectionism is best conceptualized as a multidimensional construct in which perfectionistic concerns and strivings have been identified as two higher‐order dimension of the perfectionism trait (Flett & Hewitt, 2016). Perfectionistic concerns, refer to concerns over making mistakes, fear of adverse reactions from others, and the consequences of failing to achieve high standards (Hill, 2016). The second dimension, perfectionistic strivings, comprise the strive for flawlessness and setting very high personal performance standards (Gotwals et al., 2012). Postulated by Flett and Hewitt (2016), athletes revealing perfectionistic tendencies are instilled with a win‐at‐all‐costs mentality. Therefore, athletes high in perfectionism may do whatever it takes to win including engaging in the use of doping.

Previous research has examined the link between perfectionism and doping. In a study by Jowett et al. (2023), athletes' levels of perfectionistic concerns were positively related to doping willingness. Additionally, previous research has also provided support for the positive link between perfectionistic concerns and prodoping attitudes (Madigan et al., 2020; Wang et al., 2020), suggesting that athletes concerned about making mistakes may believe that the benefits of using prohibited substances outweigh the drawbacks. There is also evidence suggesting that athletes driven by perfectionistic concerns more frequently exhibit psychological maladjustments, such as fear of failure, worry, anxiety, making this dimension of perfectionism clearly maladaptive for athletes (Hill et al., 2018).

Contrary to perfectionistic concerns, perfectionistic strivings have not been related to doping willingness (Jowett et al., 2023). Instead, a negative association with attitudes favoring doping has been shown (Madigan et al., 2016; Wang et al., 2020), echoing previous reviews which concede that perfectionistic strivings among athletes are more mixed and ambiguous (see Gotwals et al., 2012; Hill et al., 2018). Hence, it is unlikely that perfectionistic strivings are implicated in cheating behaviors, such as doping, but instead may be protective in this regard. Taken together, previous research suggests that perfectionistic concerns may exacerbate the relationship between performance climate and doping attitudes, such that this relationship may become stronger as athletes are high in their concerns. Conversely, perfectionistic strivings may strengthen the positive relationship between mastery climate and attitudes disregarding doping.

### 1.4 The present study

Performance‐ and mastery‐oriented climates are believed to differentially influence athletes' decision to dope (Guo et al., 2021; Kavussanu et al., 2020), and moreover, athletes' attitudes toward doping may function as a mediator in this relationship (Allen et al., 2015; Chan et al., 2015). Furthermore, perfectionistic concerns are likely to exacerbate the effects of performance climate on doping attitudes, making concerned athletes more susceptible do doping temptation. This may especially be the case if the athlete is placed under extreme pressures from either themselves or others to be perfect, and thus concerned about making mistakes. Conversely, perfectionistic strivings are more likely to strengthen the protective effects of mastery climate on doping attitudes, thereby reducing athletes' temptation to partake in doping. However, to our knowledge, no study has examined the path between motivational climate, attitudes, and doping temptation while exploring the moderating role of perfectionism. To gain a greater understanding of the psychosocial risk and protective factors associated with young athletes' doping temptation, it is important to examine these relationships. Therefore, the aim of the present study was to investigate how the motivational climate relates to doping temptation and to explore the moderating role of perfectionism.

Research into the psychological mechanisms related to doping has traditionally relied on self‐reports from the athletes themselves. However, due to the difficulty of obtaining reliable information on doping via direct questioning, several studies have utilized indirect measures (Hurst et al., 2022; Kavussanu et al., 2020). These measures aim to reveal athletes' true thoughts and rationalizations about doping and are thus used as proxy indicators for doping. One such measure is situational temptation, defined as an athletes' temptation to use prohibited substances and methods in hypothetical situations (Barkoukis et al., 2014; Lazuras et al., 2015).

Previous research has identified strong relationships between situational temptation and attitudes toward doping (Lazuras et al., 2015) as well as normative beliefs about doping and intentions to engage in doping (Barkoukis et al., 2014; Lazuras et al., 2015). Furthermore, due to its conceptualization, situational temptation has been employed as both an independent and dependent variable (Barkoukis et al., 2014; Lazuras et al., 2015). However, given that situational temptation pertains to the inclination to use doping under specific circumstances, one might argue that its conceptualization aligns more closely with external rather than internal control mechanisms. Hence, situational temptation could serve as a prominent and valid indicator for doping temptation.

**FIGURE 1:**
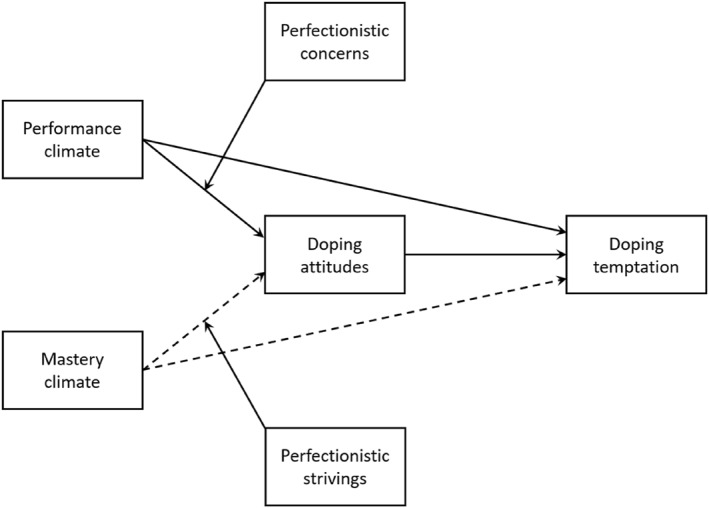
The hypothesized model of the relationships between motivational climate, attitudes toward doping, perfectionism, and doping temptation. The solid lines represent positive relationships and the dashed lines represent negative relationships. For the ease of presentation, the figure does not include the sex variable, which was included in the analysis as a covariate.

Based on the existing evidence, we hypothesized that performance climate would be positively related to doping temptation both directly and indirectly via doping attitudes (see Figure 1). Conversely, a mastery climate would be negatively related to doping temptation both directly and indirectly via doping attitudes (Allen et al., 2015; Guo et al., 2021). We also hypothesized that perfectionistic concerns would moderate the indirect relationship between performance climate and doping temptation via attitudes such that the relationship would be stronger for those with high levels of perfectionistic concerns (Madigan et al., 2020). Conversely, perfectionistic strivings would moderate the indirect relationship between mastery climate and doping temptation via attitudes such that the relationship would be stronger for those with high levels of perfectionistic strivings (Hardwick et al., 2022). Finally, considering the variations in doping behaviors between males and females, which indicate that males are more likely to reported more permissive attitudes toward doping compared to females, we included sex as a covariate (Ntoumanis et al., 2024).

## 2 MATERIALS AND METHODS

### 2.1 Participants

The sample size was estimated using a sample size calculator for multiple regression, which recommended a minimum of 103 participants to reach a power level of 0.8 to detect an anticipated effect size of 0.15 at an alpha level of 0.05, with six variables (Soper, 2018). Using a cross‐sectional design, participants (*N* = 420) aged 16–18 years (mean age = 16.94 and standard deviation [SD] = 0.81) were recruited from five Norwegian sport academy high schools that offer extracurricular, high‐level training, and specialization for youth athletes. Females comprised 50% (*n* = 209) of the sample, males comprised 45% (*n* = 188), and 5% (*n* = 23) were missing. The participants represented 19 sports, with the majority competing in handball (21%), football (19%), and ice hockey (12%). They reported having participated in organized training sessions in their sport for an average of 9.67 years (SD = 3.19) and spent an average of 14 h per week training in their sport. The prestigious sports academies were regulated by competitive auditions and offered both acceleration and enrichment in the chosen sport. Hence, those individuals attending these academies were among the most ambitious and talented athletes in their age group.

### 2.2 Procedure

The Norwegian Center of Research Data approved the project prior to its commencement (reference number 283647). After obtaining ethical clearance from the University Ethics Committee of the first author's local institution, we recruited the study participants through a dialog with the leaders of the specialized sporting schools and their respective coaches. Participants were first presented with information about the study, its purpose, and were informed that all data would be anonymous and confidential. After consenting, the participants completed the measures described below using the SurveyXact digital tool (Ramboll, 2023), which stores the data on an encrypted server. The data collection took place during November and December 2022 in Norway.

### 2.3 Measures

All the measures were administered in the Norwegian language, following the translation‐back‐translation procedure from English (Harkness, 1999).

#### 2.3.1 Perceived motivational climate

The participants' perceptions of the motivational climate were measured with a short version of the Perceived Motivational Climate in Sport Questionnaire (PMCSQ‐2; Newton et al., 2000). Participants were asked to think about what it had usually been like on their team; seven items assessed perceived performance climate (e.g., “the coach devotes most of his/her attention to the best players”), tapping into three subscales including intrateam member rivalry, punishment for mistakes, and unequal recognitions lower‐order factors, and nine items (e.g., “my coach made sure players felt successful when they improved”) captured the subscales of cooperative learning, important role, and effort/improvement encompassing perceptions of a mastery climate. Participants indicated their level of agreement on a five‐point scale (from 1 = strongly disagree to 5 = strongly agree). Both subscales demonstrated good internal reliability with McDonald's omega coefficient of 0.86 and 0.86 for the performance and mastery climate (Hayes & Coutts, 2020). The short version of PMCSQ‐2 has also been successfully used in previous research including the Norwegian context (Appleton et al., 2016; Gjesdal et al., 2019). A mean score was calculated for both performance and mastery climate subscales, with higher scores indicating stronger perceptions of performance climate or mastery climate, respectively. The same procedure was followed for all scales.

#### 2.3.2 Attitudes toward doping

Based on Ajzen's (1991) guidelines, we assessed participants' attitudes toward doping by tapping into their positive and negative evaluations of doping use. WADA's (2021) definition for doping was provided including specific examples of prohibited substances (i.e., doping is defined as the occurrence of one or more violations of the antidoping rules in which athletes make use of substances and/or methods included on the Prohibited List in or out of competition. Examples of such prohibited substances are hormones, Anabolic–androgenic steroids, and amphetamines). The measure consisted of a stem proposition (“The use of doping substances to enhance my performance this season is …”) followed by four semantic differential evaluative adjectives (bad/good, useless/useful, harmful/beneficial, and unethical/ethical) scored on a seven‐point scale. To enhance the internal consistency of this measure (i.e., Omega = 0.59), one item concerning the ethical evaluation of using doping substances was removed (i.e., “The use of doping substances to enhance my performance this season is *unethical/ethical*”); thus, the index of attitudes toward doping comprised the mean score of three items. Higher scores indicated more positive attitudes toward doping use (Omega = 0.67). Acceptable values for estimating internal reliability are normally above 0.70 (DeVellis, 2017). However, according to Hair et al. (2018), values near 0.60 could be regarded as acceptable for short scales that have fewer than 10 items. The scale has been shown to be reliable and valid in several studies related to doping behaviors including the Norwegian context (Kristensen et al., 2024; Lazuras et al., 2015).

#### 2.3.3 Perfectionism

The participants' perfectionistic concerns and strivings were measured with a contextualized version of the Frost Multidimensional Perfectionism Scale‐Brief (F‐MPS‐Brief; Burgess et al., 2016). Four items assessed perfectionistic concerns (e.g., “The fewer mistakes I make during training and competitions, the more other athletes will like me”) and four items captured perfectionistic strivings (e.g., “I set higher goals for myself in my sport than most other athletes”). Participants responded on a five‐point scale (from 1 = totally disagree to 5 = totally agree), with higher scores indicating either greater perfectionistic concerns (Omega = 0.78) or greater strivings (Omega = 0.82). The F‐MPS‐Brief is a substantial leaner yet high‐performing assessment tool for intrapersonal perfectionism and has been shown to be reliable and valid (Woodfin et al., 2020).

#### 2.3.4 Situational temptation

We measured situational temptation using the scale derived from Lazuras et al. (2015), which reflected the temptation to dope under specific circumstances in which athletes might get involved. Participants were asked to read the statements carefully while thinking about themselves. The stem proposition started “How much would you be tempted to use doping substances to enhance your performance this season?” followed by five prospective situations: “when your coach suggests so,” “when you believe that most colleagues of yours use doping substances,” “when you were told to enhance your performance,” “when you prepare for an important game/competition,” and “when feeling disadvantaged.” The responses were recorded on a five‐point scale (from 1 = not at all tempted to 5 = very much tempted), and higher scores indicated greater temptation to dope (Omega = 0.87). Conversely, a lower score denoted a greater capability to resist the temptation of using prohibited substances. In the current study, situational temptation was used as an indicator of doping temptation and thus employed as a proxy for doping behavior (i.e., dependent variable). The scale has been shown to be valid in the Norwegian sport context (Kristensen et al., 2023).

## 3 DATA ANALYSIS

IBM SPSS Statistics version 28.0 (Armonk, NY: IBM Corp) was used to compute the descriptive statistics, correlations, and regression analyses. To address the aim of our study, a conditional process analysis was considered the appropriate statistical method (Hayes, 2017). We used PROCESS v4.0 for SPSS macro (model 7) to simultaneously test the direct and indirect effects of the motivational climate on doping temptation while also including the moderating role of perfectionism. Specifically, PROCESS estimates different conditional values of the moderator for the indirect effect, and moderated mediation is tested at three levels of the moderator: one SD below the mean (i.e., low), the mean (i.e., moderate), and one SD above the mean (i.e., high). For example, if perfectionistic concerns moderate the indirect relationship between performance climate and doping temptation via attitudes, the strength and/or direction of this indirect relationship will change depending on the perfectionistic concerns score (Hayes, 2017).

Hayes (2009) recommends a bias‐corrected bootstrapping technique for estimating indirect effects and conditional indirect effects. Compared to other mediation methods, bootstrapping has been found to be more robust to abnormal distribution and tends to have greater power to detect significant effects while allowing for the control of covariates. To reveal the conditional indirect effect, PROCESS produces point estimates and bias‐corrected confidence intervals (CIs) for the hypothesized conditional indirect effect and point estimates of the remaining direct effect. Conditional indirect effects that do not include zero between the lower and upper bounds (i.e., 95% CI) demonstrate significant moderated mediation. Prior to conducting the analysis, the variables were mean‐centered using the statistical technique provided in SPSS, which involves converting them into z‐scores. This step was taken to enhance the interpretation of relationships between variables and reduce potential multicollinearity (Hayes, 2017).

## 4 RESULTS

### 4.1 Preliminary analysis

Little's Missing Completely at Random test identified 12 (2.9%) participants with data missing completely at random (chi‐squared = 34.83, df = 48, and *p* = 0.92). Missing data were handled using listwise deletion including only participants with complete data for analyses (i.e., *N* = 408). This approach was considered appropriate, as it produced a common set of cases for all analyses (Enders, 2010). Furthermore, the data violated the assumption of normality, with the symmetry of the distribution varying across the study variables, as indicated by skewness (range −1.20–4.40) and kurtosis (range −0.65–24.99). The abnormal distributed data were handled using bootstrapping in all analyses (i.e., bootstrapping was set at 10,000 samples) as advised by previous researchers (Ng & Lin, 2016).

In addition to assessing normality, SPSS identified a few potential outliers on mastery climate, attitudes toward doping, and doping temptation. However, in line with Sullivan and colleagues' (2021) reflections concerning the valid indicator for outlier labeling, we critically examined only those outliers that qualified for the third interquartile range. Specifically, two outliers were identified on mastery climate, whereas 11 and 16 cases were identified on attitudes toward doping and doping temptation, respectively. Given the context of competitive sports and the self‐presentational biases associated with self‐reports on doping behaviors (Backhouse et al., 2013), we did not find these outliers particularly concerning. Hence, we did not remove any of the identified outliers prior the main analyses, as they may represent natural variation in the population and, as such, provide new insights.

### 4.2 Descriptive statistics, internal consistency, and zero‐order correlations

Descriptive statistics, McDonald's omega coefficients, and zero‐order correlations for all measures are reported in Table 1. As indicated by the mean values, the table shows that the athletes reported scores above the scale's midpoint for performance climate, mastery climate, perfectionistic concerns, and perfectionistic strivings, whereas the scores for doping attitudes and doping temptation were below the midpoint. Zero‐order correlations showed that performance climate, doping attitudes, and perfectionistic concerns were positively associated with doping temptation. Mastery climate and perfectionistic strivings were not significantly related to doping temptation.

**TABLE 1:** Descriptive statistics, internal consistency, and zero‐order correlations (*N* = 408).

Variable	Mean (SD)	Omega [95% CI]	1	2	3	4	5	6	7
1. Performance climate	2.82 (0.87)	0.86 [0.84, 0.88]	‐						
2. Mastery climate	3.89 (0.64)	0.86 [0.81, 0.89]	−0.21^a^	‐					
3. Doping attitudes	1.48 (0.89)	0.67 [0.57, 0.78]	0.19^a^	−0.03	‐				
4. Perfectionistic concerns	2.81 (0.99)	0.78 [0.73, 0.81]	0.33^a^	−0.08	0.16^a^	‐			
5. Perfectionistic strivings	3.65 (0.86)	0.82 [0.78, 0.85]	0.15^a^	0.18^a^	0.06	0.31^a^	‐		
6. Doping temptation	1.21 (0.50)	0.87 [0.75, 0.92]	0.22^a^	−0.01	0.37^a^	0.20^a^	0.08	‐	
7. Sex	0.45 (0.50)	n/a	0.03	−0.08	0.27^a^	0.01	0.20^a^	0.02	‐

*Note*: Bootstrapped descriptive statistics and zero‐order correlation coefficients. Number of bootstrapped resamples = 10,000. Bias‐corrected 95% confidence intervals including lower and upper limits for closed‐form McDonald's omega in brackets. The possible range of responses is 1–5 for all variables except attitudes (1–7). Sex was treated as a dummy variable and coded as 0 = females and others and 1 = males.

^a^ *p* < 0.01 (two‐tailed).

### 4.3 Main analysis

Aligned with our hypothesized model, we tested two equivalent sequences between motivational climate, attitudes, and doping temptation while including the moderating role of perfectionism. To ensure the direct effect of performance and mastery climate on doping temptation that is unique of the other, we controlled for the influence of one predictor (e.g., mastery climate) while examining the direct effect of the other predictor (e.g., performance climate).

In the first moderated mediation analysis, performance climate was directly related to doping temptation (*b* = 0.16, 95% CI_c’_ = 0.07–0.26, and *p* < 0.001). Moreover, performance climate was also associated with positive attitudes toward doping (*b* = 0.15, 95% CI_a1_ = 0.05–0.24, and *p* < 0.01), which in turn, independently predicted doping temptation (*b* = 0.36, 95% CI_b1_ = 0.05–0.24, and *p* < 0.01). The indirect effects revealed that doping attitudes mediated the relationship between performance climate and doping temptation (*b* = 0.05 and 95% CI_ab_ = 0.01–0.12). Finally, after controlling for the possible nonindependence of sex on attitudes and doping temptation, results suggested that sex accounted for variation in attitudes (*b* = 0.56, 95% CI_a4_ = 0.34–0.74, and *p* = 0.001) but no doping temptation. The latter influence of sex indicates differences in males and females' attitudes toward doping, with males reporting more positive attitudes toward doping compared to females.

The index of moderated mediation was significant (index = 0.05 and 95% CI = 0.01–0.14) suggesting that the indirect relationship between performance climate and doping temptation via attitudes varies as a function of perfectionistic concerns. Perfectionistic concerns (*b* = 0.11, 95% CI_a2_ = 0.02–0.21, and *p* < 0.05) and the interaction between performance climate and perfectionistic concerns (*b* = 0.13, 95% CI_a3_ = 0.05–0.22, and *p* < 0.01) were both positive significant predictors of attitudes toward doping. To probe the significant interaction, conditional indirect effects of performance climate on doping temptation via attitudes were tested at three levels of perfectionistic concerns; low (*W* = −1.00), moderate (0.00), and high (1.00).

**FIGURE 2:**
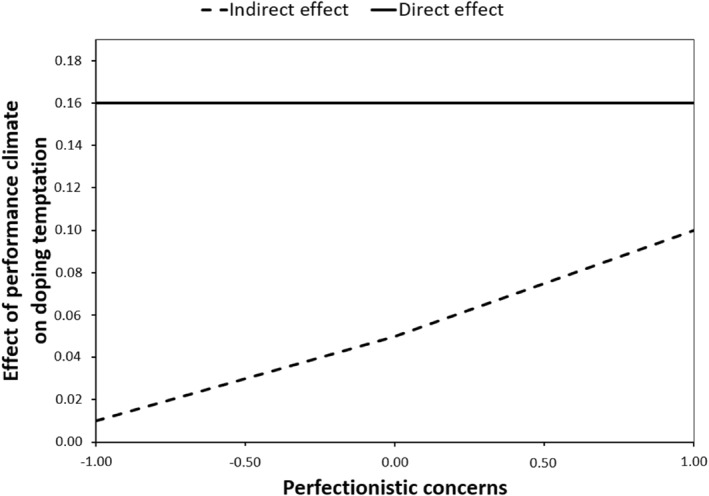
A visual representation of the conditional indirect and the direct effect of performance climate on doping temptation, with the indirect effect operating through attitudes toward doping. The slope of the line illustrates how the indirect effect varies with changes in perfectionistic concerns by one unit.

These tests showed that the indirect relationship of performance climate and doping temptation was significant only when perfectionistic concerns were moderate (effect = 0.05 and 95% CI = 0.01–0.12) and high (effect = 0.10 and 95% CI = 0.02–0.25) but not when they were low (effect = 0.01 and 95% CI = −0.05 to 0.04). Pairwise contrasts between conditional indirect effects revealed significant difference among low, moderate, and high perfectionistic concerns, suggesting that each conditional indirect effect is significantly different from the others. A visual depiction of the conditional indirect effect of performance climate on doping temptation via attitudes can be seen in Figure 2. The slope of the line illustrates how the indirect effect varies with changes in perfectionistic concerns by one unit.

In the second moderated mediation analysis, mastery climate was not directly related to doping temptation (*b* = 0.03, 95% CI_c’_ = −0.06–0.13, and *p* = 0.47) nor was mastery climate related to attitudes toward doping (*b* = 0.04, 95% CI_a1_ = −0.06–0.13, and *p* = 0.45). When exploring the moderating role of perfectionistic strivings in the indirect relationship between mastery climate and doping temptation via attitudes, perfectionistic strivings (*b* = −0.02, 95% CI_a2_ = −0.12 to 0.08, and *p* = 0.66) and the interaction between mastery climate and perfectionistic strivings (*b* = 0.01, 95% CI_a3_ = −0.05–0.08, and *p* = 0.70) were nonsignificant, suggesting that perfectionistic strivings did not moderate the indirect relationship between mastery climate and doping temptation.

## 5 DISCUSSION

The purpose of the present study was to examine whether the motivational climate was directly and indirectly (via attitudes toward doping) related to doping temptation and whether perfectionism moderated the motivational climate–doping attitudes relationship. The target group of young aspiring athletes is of particular importance for doping prevention due to their susceptibility to accept and take behavioral risks in the service of attaining high‐level performance (Kristensen et al., 2022).

### 5.1 Motivational climate and doping temptation

In line with our hypothesis, performance climate predicted doping temptation both directly and indirectly via attitudes toward doping. This finding aligns with the idea of a socialization effect (Ames, 1992) suggesting that a sporting environment characterized by social comparison and being the best might enable athletes to engage in doping by eliciting more favorable attitudes toward prohibited substances. Previous studies have also shown that performance climate positively predicts doping likelihood (Kavussanu et al., 2020), doping intentions (Guo et al., 2021), and dysfunctional behavioral patterns (Ommundsen et al., 2003). It appears that athletes are more likely to be tempted to dope when they perceive the coach as emphasizing normative success.

The finding also highlights the facilitating role of attitudes toward doping—a psychological tendency expressing varying degrees of favorability or disfavor toward doping use (Eagly & Chaiken, 1993). Attitudes toward doping have been associated with various doping behaviors, including doping susceptibility (Barkoukis et al., 2014) and doping intentions (Lazuras et al., 2015), thus believed to influence athletes' decision about whether to engage in doping (Barkoukis et al., 2014). Due to their fluctuation and adaptation to environmental demands, attitudes may serve as a mediator in the relationship between performance climate and doping temptation. Specifically, a performance climate might increase athletes' doping temptation by eliciting a stronger belief that using prohibited substances would lead to more positive than negative consequences (Eagly & Chaiken, 1993).

Contrary to theoretical postulates (Ames, 1992) and previous research (Allen et al., 2015; Guo et al., 2021), the results showed that mastery climate was not related to doping temptation or attitudes toward doping. Previous research has considered mastery climate as a situational factor that could potentially shield athletes from doping (Allen et al., 2015). One possible explanation for this discrepancy could be that a mastery climate is typically considered motivationally and behaviorally adaptive, activating psychological mechanisms leading athletes to proactive behavioral patterns, such as moral reasoning and fair play (Ommundsen et al., 2003). However, it is plausible that such a climate may not be able to take the edge of environmental pressure emphasizing social comparison standards for success, thereby ineffective to reduce athletes' temptation to make use of doping.

It is worth noting that there were differences in attitudes toward doping between males and females, with males exhibiting more favorable attitudes toward doping compared to females. This suggest that men may be more inclined toward doping, potentially influencing their judgments regarding the use of such prohibited performance‐enhancing substances. In a recent systematic review, Gleaves et al. (2021) reported disparities in attitudes toward doping between males and females but expressed caution in drawing conclusions. Nevertheless, the findings of the current study highlight that doping prevention programs aimed at reducing prodoping attitudes among young athletes would benefit from considering gender differences (Pöppel & Büsch, 2022).

### 5.2 Perfectionism and doping temptation

In line with our hypotheses concerning the moderating role of perfectionism, perfectionistic concerns moderated the indirect relationship between performance climate and doping temptation via attitudes. That is, the indirect relationship between performance climate and doping temptation via attitudes was stronger among athletes with greater concerns over mistakes, and moreover, statistically significant only among athletes who are moderate or high in their perfectionistic concerns. For athletes low in perfectionistic concerns, the indirect relationship seemed to dampen. Hence, the results suggest that athletes' overly critical evaluation of themselves might heighten their susceptibility to doping by exacerbating the effects of their perceptions of a performance climate on their attitudes toward doping. That is, concerns about failure or making mistakes during competition exacerbate the psychological maladjustments of performance climate on attitudes, making these attitudes more permissive to doping. As such, perfectionistic concerns may potentially serve as a risk factor for doping temptation (Hardwick et al., 2022; Madigan et al., 2020).

The findings of the current study add to previous research on perfectionism and doping, in which perfectionistic concerns were found to be positively associated with prodoping attitudes (Madigan et al., 2020; Wang et al., 2020). Additionally, our findings also bring support to the assumption that perfectionistic concerns may moderate the impact of environmental influences on these attitudes (Conner & Sparks, 2015). Specifically, our study suggest that athletes' perceptions of a performance climate can lead them to view doping as an acceptable means to enhance their sports performance, thereby increasing their temptation to dope. This inclination is particularly notable among athletes who are concerned about making mistakes and experiencing failure. These findings are interesting, as they indicate that athletes may resort to extreme measures to meet with environmental expectations and performance pressures and underline the heightened sensitivity of perfectionistic concerned athletes.

Contrary to expectations and theoretical postulates (Flett & Hewitt, 2016), perfectionistic strivings did not moderate the indirect relationship between mastery climate and doping temptation via attitudes. Our results suggest that perfectionistic strivings may not be related to doping temptation and prodoping attitudes, and moreover, may not moderate the mastery climate–attitudes relationship. However, these findings echo previous work illustrating the complexity of perfectionistic strivings and its relationship with doping attitudes. In particular, Hardwick et al. (2022) reported a nonsignificant relationship between perfectionistic strivings and attitudes in favor of doping, whereas Madigan et al. (2016) found a negative association between the two. It appears that athletes' striving tendencies toward achievement might not be a perfectionistic disposition that deters them from the misuse of engaging in prohibited substances. However, there may be other mechanisms by which perfectionistic strivings influence the decision to dope (Hardwick et al., 2022). Hence, more research is needed to explore the relationship between perfectionistic tendencies and doping antecedents such as liberal attitudes toward doping. Taken together, our findings suggest that athletes' temptation to engage in doping may be influenced not only by their perceptions of a performance climate but also by their attitudes toward doping. Moreover, athletes revealing perfectionistic tendencies of being concerned may be particularly tempted to engage in doping.

## 6 IMPLICATIONS FOR PRACTICE

The present findings extend the existing literature on doping and offer important practical recommendations. First and foremost, coaches need to recognize their influential role in shaping athletes' attitudes and behaviors. In their interaction with athletes, coaches should downplay the importance of winning, competition, and intrateam rivalry. Given the performance‐oriented nature of sports, where winning is often highly emphasized, coaches are also encouraged to be more explicit and braver in balancing performance demands against athletes' health and psychosocial well‐being. In terms of educational interventions aimed at preventing doping, the present findings suggest that efforts to change the coach‐created performance climate and attitudes toward doping may be worthy to further explore. These targeted interventions should also consider gender differences and perfectionistic tendencies (Madigan et al., 2020; Pöppel & Büsch, 2022).

## 7 LIMITATIONS AND FUTURE RESEARCH

The current study has some limitations, and the following should be considered when interpreting the study findings. First, the study relied on self‐reports from the athletes themselves, making data vulnerable to self‐presentational biases suggesting that the participants may have been inclined to present themselves in a favorable light (Backhouse et al., 2016). To mitigate social desirability, we implemented several strategies. These included ensuring anonymity of response, emphasized confidentiality, and utilizing indirect measures (Petróczi, 2016). Second, the cross‐sectional design precludes assertions about causal relationships. Even though we hypothesized that opposing motivational climate would differentially predict doping temptation, with perfectionism set to operate as a moderator, it is also possible that differential motivational climates act to moderate the relationship between perfectionism and doping temptation (Kavussanu et al., 2020). Third, the reliability of the attitudes toward the doping scale was somewhat low, warranting caution when interpreting findings involving this subscale. Finally, an adapted version of the F‐MPS‐Brief (Burgess et al., 2016) was used in the current study to assess athletes' perfectionism in sport. However, compared to domain‐specific measures, such as the Sport Multidimensional Perfectionism Scale (Dunn et al., 2011), the F‐MPS‐Brief may have some shortcomings when it comes to capturing individual differences in perfectionism in sport.

To further advance our understanding of adolescent athletes' doping temptation, future research should broaden their scope to include other intrapersonal variables (e.g., moral identity and moral values) and investigate at‐risk populations (e.g., athletic discipline, competition level, and age group). Given the complex interplay between the personal factors of the athletes and the contextual factors summarized from athletes' self‐described perceptions of the sporting environment, classification tree analysis could be a prominent analysis tool for identifying those at‐risk for turning to doping (Lemon et al., 2003). Finally, to provide more robust evidence for the directions of causality, future research should examine if the explanatory pathways identified in the present study replicate in longitudinal, multiwave studies, and whether performance climate operate as a predictor or moderator (VanderWeele, 2015).

## 8 CONCLUSION

A social cognitive approach helps explain the psychosocial conditions underlying athletes' temptation to dope. Athletes who perceive their sport environment as performance oriented may be more tempted to dope due to their attitudinal belief that using prohibited performance‐enhancing substances would lead to more positive than negative consequences. This inclination is particularly notable among athletes who are concerned about making mistakes. Conversely, athletes' perceptions of a mastery‐oriented climate and their perfectionistic striving tendencies do not appear to be related to their temptation to dope, thereby seemingly being ineffective in enhancing our understanding of potential factors that could protect young athletes from doping temptation.

## 9 ENDNOTE

We conducted confirmatory factor analysis using *M*plus (Muthén & Muthén, 1998–2017) to examine the factorial structure of the attitudes scale. Initially, we assessed the scale's original four indicators using the robust maximum likelihood estimation method (van Zyl & ten Klooster, 2022). We relied on common good‐ness‐of‐fit indices, including comparative fit index (CFI), Tucker–Lewis's index (TLI), root mean square error of approximation (RMSEA), and standardized root mean square residual (SRMR), which indicated poor fit for the single factor model (i.e., RMSEA = 0.30, 90% CI: [0.24, 0.36], CFI = 0.85, TLI = 0.54, and SRMR = 0.11). After inspecting item correlations, an alternative model was tested in which the first and fourth items were specified to correlate with each other using the WITH command in *M*plus. This adjustment resulted in a good fit (i.e., RMSEA = 0.01, 90% CI: [0.00, 0.11], CFI = 1, TLI = 1, and SRMR = 0.01).

## CONFLICT OF INTEREST STATEMENT

The authors declare that they have no conflicts of interest.

## Data Availability

The data supporting this study’s findings are available on request from the corresponding author.
